# AVA Virtual Spring Meeting 29th March 2021

**DOI:** 10.1177/20416695211039080

**Published:** 2021-08-31

## GJ Burton Memorial Lecture

## Spectral slope in vision and art

**George Mather**

School of Psychology, University of Lincoln, Lincoln, UK

gmather@lincoln.ac.uk

In 1987 Geoffrey Burton and Ian Moorhead published an influential paper on the image statistics of natural scenes, which showed that Fourier amplitude is inversely proportional to spatial frequency: Each doubling in frequency is associated with a halving in amplitude (1/f scaling). The visual system could exploit this statistical regularity to optimise processing so that it matches the properties of natural scenes, and there is psychophysical evidence that this is the case. 1/f scaling in processing has implications for visual art: When artists create paintings, they may regularise the statistics of their paintings so that they conform to 1/f scaling. In this talk I will review some of the research I have conducted to investigate 1/f scaling in visual art. One study involves comparisons between artworks and corresponding photographs of the same scenes. How do their spectral slopes compare? Another study measures the visual statistics of over 500 artworks created during the last 500 years, to track the stability of visual statistics over a time period that spans multiple artistic periods and styles. The latter study indicates that in a statistical sense the most significant turning point in art history took place around 1880. Geoffrey Burton and Ian Moorhead’s work on image statistics turns out to have applications in the most unexpected places.

## Talks

## Attention to Colour: Spotlight in Hue Space

**Jasna Martinovic^1,2^, Antoniya Boyanova^1^ and Søren K. Andersen^1^**

^1^School of Philosophy, Psychology and Language Sciences, University of Edinburgh, Edinburgh, UK

^2^School of Psychology, University of Aberdeen, Aberdeen, UK

j.martinovic@ed.ac.uk

Biological systems must allocate limited perceptual resources to relevant elements in their environment. In vision, this can be achieved by attending to a spatial location or a defining feature. Spatial attention can be conceived as a spotlight in 2D space, whose focus can differ in broadness and concentration. Could attention to colour also be distributed as a spotlight that highlights an area of colour space? We examined this in an experiment that used multi-coloured displays with 4 overlapping random dot kinematograms (RDKs) that differed only in hue. We manipulated (1) requirement to focus attention to a single colour (red or green) or divide it between two colours (red and green); (2) distances of distractor hues from target hues, resulting in three different contexts which differed in proximity between the two distractors and the two targets. We conducted a behavioural experiment and an electroencephalographic experiment, in which each colour was tagged by a specific flicker frequency and driving its own steady-state visual evoked potential. Behavioural and neural indices of attention showed several major consistencies. Concurrent selection of red and green reduced the efficiency of target enhancement while additively combining the false alarms elicited in the corresponding single target conditions. This could result from the combination of two spotlights of unchanged broadness but reduced concentration. Based on this conceptual similarity, we conclude that attentional modulation of signals in biological systems is likely to be implemented using the same domain-general mechanisms, which are well-described with a spotlight metaphor.

## Skin Colour is a Culturally-Specific Cue for Attractiveness, Healthiness and Youthfulness in Chinese and Caucasian Observers

**Yan Lu^1^, Jie Yang^1^, Kaida Xiao^1^, Michael Pointer^1^, Changjun Li^2^ and Sophie Wuerger^3^**

^1^School of Design, University of Leeds, Leeds, UK

^2^School of Computer Science and Software Engineering, University of Science and Technology Liaoning, Anshan, China

^3^Department of Psychology, University of Liverpool, Liverpool, UK

sdyl@leeds.ac.uk

Facial skin colour signals information about an individual and plays an important role in social interactions and mate choice, due its putative association with health, attractiveness, and age.

The current study used 80 calibrated, high-resolution, non-manipulated images of real human faces, either Chinese and Caucasian, which were rated in terms of attractiveness, healthiness, and perceived age by observers of the same or the other ethnicity. To elucidate the associations between skin colour and the these perceptual ratings and whether these associations are modulated by observer or image ethnicity, a linear mixed-effect model was setup with skin lightness (L*; CIELAB), redness (a*) and yellowness (b*), observer and image ethnicity as independent variables and perceived attractiveness, healthiness and estimated age as dependent variables.

We found robust positive associations between facial skin lightness (L*) and attractiveness, healthiness, and youthfulness, but only when Chinese observers judge facial images of their own ethnicity. Caucasian observers, on the other hand, associated an increase in yellowness(b*) with greater attractiveness and healthiness in Chinese facial images. We find no evidence that facial redness is positively associated with these attributes; instead an increase in redness (a*) is associated with an increase in the estimated age of Caucasian facial images.

We conclude that observers of both ethnicities make use of skin colour and lightness to rate attractiveness, healthiness, and perceived age, but to a lesser degree than previously thought. However, these coloration cues are not universal and are utilized differently within the Chinese and Caucasian ethnic groups.

## Parallel popout: further confirmation of the V1 Saliency Hypothesis (V1SH)

**Li Zhaoping**

University of Tübingen, and Max Planck Institute for Biological Cybernetics, Tübingen, Germany

li.zhaoping@tuebingen.mpg.de

Finding a target among uniformly oriented non-targets is typically faster when this target is perpendicular, rather than parallel, to the non-targets. Here, by exploiting the properties of saliency computations in primary visual cortex (V1), I demonstrate a special case when exactly the opposite is true. Each item, target or non-target, comprises two disks of the same size; the centre of one disk is displaced 1.2 disk diameters from that of the other along a line defining the item’s orientation. A target has two black disks or two white disks; each non-target has one white disk and one black disk. The target is oriented 45 degree clockwise or counter-clockwise from horizontal; the non-targets are uniformly oriented either perpendicular or parallel to the target in a grey background. Unlike the target, each non-target activates a neuron in V1 more strongly when its orientation is perpendicular rather than parallel to the neuron’s preferred orientation, since the white and the black disks best activate, respectively, the on- and off- subfields of the neural receptive field (Zhaoping, L., 2020, i-Perception, 11(4):1–5.). V1 neurons are suppressed more strongly by neighbouring neurons tuned to similar rather than dissimilar orientations. Thus, a target parallel (rather than perpendicular) to the non-targets evokes a higher V1 response and, according to V1SH, is more salient. Our behavioural confirmation of faster search in this condition supports V1SH’s proposal that V1 is the neural basis for saliency of exogenous attentional selection (Zhaoping, L., 2002, Trends in Cognitive Sciences 6(1):9-16).

## Behavioural performance improvement in visuomotor learning correlates with functional and microstructural brain changes

**E. Aloufi^1^, Fiona .J. Rowe^2^ and Georg F. Meyer^1^**

^1^Department of Psychology, University of Liverpool, Liverpool, UK

^2^Institute of Population Health, University of Liverpool, Liverpool, UK

athab327@liverpool.ac.uk

A better understanding of practice-induced functional and structural changes in our brains can help us design more effective learning environments that provide better outcomes. Although there is growing evidence from human neuroimaging that experience-dependent brain plasticity is expressed in measurable brain changes that are correlated with behavioural performance, the relationship between behavioural performance and structural or functional brain changes, and particularly the time course of these changes, is not well characterised.

To understand the link between neuroplastic changes and behavioural performance, 15 healthy participants in this study followed a systematic eye movement training programme for 30 min daily at home, 5 days a week and for 6 consecutive weeks. Behavioural performance statistics and eye tracking data were captured throughout the training period to evaluate learning outcomes. Imaging data (DTI and fMRI) were collected at baseline, after two and six weeks of continuous training, and four weeks after training ended. Participants showed significant improvements in behavioural performance (faster task completion time, lower fixation number and fixation duration). Spatially overlapping reductions in microstructural diffusivity measures (MD, AD and RD) and functional activation increases (BOLD signal) were observed in two main areas: extrastriate visual cortex (V3d) and the frontal part of the cerebellum/Fastigial Oculomotor Region (FOR), which are both involved in visual processing. An increase of functional activity was also recorded in the right frontal eye field. Behavioural, structural and functional changes were correlated.

## The impact of auditory, visual and crossmodal cues on multiple object tracking

**Julia Föcker^1^, Polly Atkins^1^, Foivos-Christos Vantzos^1^, Maximilian Wilhelm^2^, Thomas Schenk^3^ and Hauke S. Meyerhoff^4^**

^1^School of Psychology, University of Lincoln, Lincoln, UK

^2^Center for Psychotherapy Research, University Hospital Heidelberg, Heidelberg, Germany

^3^Faculty of Psychology and Educational Science, Ludwig-Maximilian University Munich, Munich, Germany

^4^Leibniz-Institut für Wissensmedien, Tübingen, Germany

Jfoecker@lincoln.ac.uk

Previous research suggested that visual cues (e.g. a colour change) can improve multiple object tracking by updating the spatio-temporal information of the target (Papenmeier et al., 2014, J Exp Psychol Hum Percept Perform 40 159–171). Building up evidence on guiding visual attention by the temporal coincidence of visual changes and a tone (van den Burgh et al., 2008, J Exp Psychol Hum Percept Perform 34 1053-1065), we asked whether auditory and audio-visual cues improve tracking performance comparably to visual cues. In Experiment 1, participants tracked five out of ten moving objects within a circular area. For each target, we presented a visual cue, an auditory cue, neither, or both at the moment the target object bounced off the central border of the tracking area. We observed that auditory cues improved tracking although they were less effective than visual cues. In Experiment 2, we manipulated the number of tracked targets (3 vs. 5) in order to study whether attentional load modulates the impact of sensory cues on multiple object tracking. Most importantly, we replicated the beneficial impact of the auditory and visual cues on tracking performance. We also observed an effect of the attentional load, but no modulation of the impact of the cues by attentional load. We discuss possible training regimes in the context of therapeutic settings to restore attentional capacity, e.g. in individuals with visual deficits.

## Enlightened orientation—How dung beetles compensate for light-polluted skies

**James J. Foster^1^, Claudia Tocco^2^, Jochen Smolka^3^, Lana Khaldy^3^, Emily Baird^4^, Marcus J. Byrne^2^, Dan-Eric Nilsson^3^ and Marie Dacke^3^**

^1^Zoologie II, Würzburg University, Würzburg, Germany

^2^School of Animal, Plant and Environmental Sciences, University of the Witwatersrand, Johannesburg, South Africa

^3^Biologiska institutionen, Lund University, Lund, Sweden

^4^Department of Zoology, Stockholm University, Stockholm, Sweden

james.foster@uni-wuerzburg.de

Many night-active animals rely on compass cues in the sky to find their way, including the moon, skylight polarization pattern and the stars (Foster et al., 2018 Proc. R. Soc. Lond. B 285, 2017232). Light projected upward into the sky is scattered back downwards in the form of ‘skyglow’, which can obscure these celestial compass cues (Foster et al., 2019 J. Exp. Biol. 222, jeb188532). In this study, we investigated how light pollution, in combination with different celestial cues, affects orientation in the ball-rolling African dung beetle Scarabaeus satyrus Boheman. This nocturnal species performs a well-described orientation behaviour, typically relying on the lunar polarization pattern and the Milky Way to hold its course. We recorded orientation behaviour at a light-polluted urban site and a dark-sky rural site, under moonlit, starlit and overcast skies. Available visual information in each scene was recorded using a calibrated camera system, and processed to quantify potential compass cues. We find that vital celestial cues are obscured and degraded by skyglow, forcing these beetles to rely instead on terrestrial beacons, which would not support compass orientation at a larger scale. For the many other species of insect, bird and mammal that rely on the night sky for orientation and migration, these effects could dramatically hinder their vital night-time journeys.

## A four-dimensional space for action perception?

**Laura C. Vinton, Steven P. Tipper and Nick E. Barraclough**

Department of Psychology, University of York, York, UK

lcv503@york.ac.uk

In our social environment we evaluate the actions of other individuals from the variety of kinematics that reveal an individual’s goals and even their intentions. This range of information allows us to make efficient and appropriate behavioural and social responses. In this study, we explored the representational space underlying visual action perception, in a similar way to measurements of face trait space (Sutherland et al., 2013 Cognition 127 105-118) and the visual and haptic perception of objects (Gaißert et al., 2010 Journal of Vision 10(11) 20). We recorded 240 different actions using motion capture and used this data to animate a volumetric avatar that performed the different actions. Participants (n = 235) viewed all actions and rated (on a 1-9 Likert scale) the extent to which each action demonstrated one of 23 different action characteristics (e.g., avoiding-approaching, pulling-pushing, weak-powerful, etc.). An exploratory factor analysis was run on the average ratings to examine the latent factors that underlie visual action perception. The best fitting model was a four-dimensional oblique model. We named the factors: unfriendly-friendly, feeble-formidable, adducting-abducting, and unplanned-planned. The results suggest the first two most dominant factors, of friendliness and formidableness, might overlap with evaluation of other stimuli types (e.g., face traits) whilst the last two factors may be unique to actions.

## The effect of video-game visual training on visual function in adults with self–reported reading difficulties

**Agne Mikailionyte^1^, Barbara Pierscionek^2^, Francesca E Mackenzie^1^ and Andy Augousti^1^**

^1^Kingston University London, Kingston, UK

^2^Anglia Ruskin University, Cambridge, UK

f.mackenzie@kingston.ac.uk

Reading difficulties (RD) affect more than 6.3 million people in the United Kingdom. Previous studies have shown that 20 hours of visual training by video-gaming improves visual function in dyslexic children (Franceschini et al, 2017 Sci Rep 7:5863), more so than a year of reading therapy. It is not known whether adults may display the same improvements. We assessed the effects of video-game training on visual function in adults (age 18+ years) with self-reported RD (SSRD) and without (non-SSRD), using psychophysical tests. Non-SRRD adults with previous video-gaming experience (‘gamers’, n = 17) had generally higher contrast sensitivity (CS) compared to non-gamers (n = 9). 120-hour (n = 4) or 40-hour (n = 11) video-game training of non-gaming, non-SRRD participants using either ‘action’ or ‘casual’ games improved CS, which remained stable over at least 4 weeks (n = 5). Finally, CS improved in adults with self-reported RD (SRRD) after either 40 (n = 4) and even only 20 (n = 6) hours of video-game training. In conclusion, visual training using video-gaming improved visual function in adults with self-reported RD, which we hypothesise may be due to improved visual attention. Video game play may serve as an accessible and inexpensive therapeutic tool in alleviating self–reported RD in adults. Future research is required to assess whether visual training can improve daily reading ability in adults with RD.

## Posters

## Audio-visual Stimulation for Visual Compensatory Functions in Stroke Survivors with Hemianopia; A Systematic Review

**Kholoud Alwashmi^1,3^, Georg Meyer^1^ and Fiona Rowe^2^**

^1^Department of Psychology, University of Liverpool, UK

^2^Department of Health Services Research, primary care and mental health, University of Liverpool, UK

^3^Department of Radiology, Princess Nourah bint Abdulrahman University, KSA

K.alwashmi@liverpool.ac.uk

Hemianopia is complete or partial blindness of half of the visual fields, caused mainly by cerebral infarction. It has been hypothesized that systematic audio-visual training (AVT) of the blind hemifield can improve accuracy and search times in visual exploration, probably due to stimulation of the Superior Colliculus (SC), an important multisensory structure involved in the initiation and execution of saccades (Passamonti et al., 2009 Neuropsychologia, 47, 546-555). Our review aims at assessing and presenting evidence for effectiveness of AVT as a rehabilitation method for hemianopia patients in adulthood and childhood. The review protocol is registered with PROSPERO. A narrative synthesis of the findings is presented to highlight how AVT rehabilitation impacts on hemianopia patients including visual oculomotor function, functional ability in activities of daily living, dyslexia, visual scanning and searching tasks, maintenance of functional ability post training and the effect on brain multisensory integration by using neuroimaging. Our findings support the concept that compensatory AVT can be useful as a rehabilitation method for hemianopia. Systematic AVT may improve the processing of visual information by recruiting subcortical pathways and, because most of the stroke survivors with visual cortex damage have an intact SC, it might be possible to train use of retinotectal functions by the AVT (Dundon et al., 2015 Frontiers in Behavioral Neuroscience, 9). Nevertheless, there is a considerable lack in studies using AVT on human hemianopia patients with concurrent use of functional and structural MRI in order to identify the optimal training paradigm and the neural mechanisms underlying its effect.

## Effects of ageing on suprathreshold contrast vision

**Maliha Ashraf^1^, Sophie Wuerger^1^, Jasna Martinović^3^ and Rafał Mantiuk^2^**

^1^Department of Psychology, University of Liverpool, Liverpool, UK

^2^Dept. of Computer Science and Technology, University of Cambridge, Cambridge, UK

^3^School of Philosophy, Psychology and Language Sciences, University of Edinburgh, Edinburgh, UK

maliha.ashraf@liverpool.ac.uk

The human visual system undergoes several changes as it ages, including changes in lens density, pupil size, neuron death, etc. Some of these physiological factors affect spatio-chromatic contrast vision. While there is ample evidence to show that contrast sensitivity at threshold decreases with age particularly at high spatial frequencies and low luminance levels, studies on the effects of ageing on suprathreshold vision are relatively scarce. We investigated suprathreshold contrast matching across luminance levels (0.02 – 2000 cd/m2) for two age groups (young: < 60 yrs, old: > 60 yrs). The stimuli were Gabor patches varying in three cardinal color directions (achromatic, red-green, yellowish-violet), and 3 spatial frequencies (0.5, 1, and 2 cpd). We displayed the reference stimulus on a SDR display (fixed to 20, or 200 cd/m2) on the right, and the test stimulus on a HDR display (at one of the six tesdt luminance levels) on the left. The observers could adjust the contrast of the test stimulus to match that of the reference stimulus. Three reference stimulus contrasts (low, medium, high) were used. We found that the difference between equal-perceived contrast curves obtained from the two age groups decreased with the absolute value of reference contrast. That is, the older group overestimated the test contrast at low contrast conditions (closer to threshold). The differences between the two groups diminished for high contrast conditions. The difference between the two age groups also increased with higher spatial frequencies and lower mean luminance levels.

## The effects of noise carriers on first-order shape-from-shading depth judgements in older adults

**Hannah E Broadbent^1^, Andrew J Schofield^2^ and Harriet A Allen^1^**

^1^School of Psychology, University of Nottingham, Nottingham, UK

^2^School of Psychology, Aston University, Birmingham, UK

hannah.broadbent@nottingham.ac.uk

The study explores the effects of age on shape-from-shading sensitivity and how the use of a noise carrier can affect performance and processing time. Research has suggested that modulation of the luminance properties enables the interpretation of surface shape (Shape-from-shading; Kleffner & Ramachandran, 1999 Perception & Psychophysics 52 18-36). In addition to this, the ‘light from above’ assumption means that rotating the orientation of a luminance grating by 180° can make stimuli appear either convex or concave (Schofield et al., 2011 Vision Research 51 2317-2330). In a previous study we found that older participants were significantly impaired in completing a shape-from-shading task at higher spatial frequencies, despite being able to see the carrier. Observers aged 18-25 and 60+ took part in online experiments looking at the effect of noise carrier on accuracy and reaction time in a shape-from-shading task. Observers completed a visual search task where they made a response to a concave stimulus amongst an array of convex stimuli. The stimuli were composed of either a luminance modulation only, or a luminance modulated noise carrier. The noise carriers used were binary noise and a fine-scale isotropic noise. We found that reaction times for older adults did not significantly increase when compared to the younger adults whose performance was more negatively impacted by the use of a noise carrier. Research into this field is important in looking at how young and old adults process the perceived depth though first-order only shape-from-shading in textured stimuli.

## Viewing face space from a different angle

**Ryan Elson^1^, Michel Valstar^2^, Denis Schluppeck^1^ and Alan Johnston^1^**

^1^School of Psychology, University of Nottingham

^2^School of Computer Science, University of Nottingham

ryan.elson@nottingham.ac.uk

‘Face space’ (Valentine, 1991 The Quarterly Journal of Experimental Psychology Section A 43 161-204) has been a highly influential account of how faces might be cortically represented. Recently, it has been proposed that rather than having just one overarching face space, we also have separate identity-specific spaces for familiar faces (Burton, et al., 2016 Cognitive Science 40 202-223), encompassing within-person variation caused by pose, illumination, and expression changes. How face spaces encompass changes in pose has not yet been fully established. Automatic face analysis systems mostly deal with pose by either aligning to a canonical frontal view or by using separate view-specific models. There is little evidence that the brain possesses an internal 3D model for ‘frontalising’ faces, therefore here we investigate how changes in view might be processed in a unified multi-view face space based on using a few prototypical 2D views. We investigate the functionality and biological plausibility of various identity-specific faces spaces, created using principal components analysis (PCA), that allow for different views to be reconstructed from single-view video inputs of actors speaking. We also compare different methods of representing views within different spaces and subspaces. If we collate views captured simultaneously into multi-vectors, we can recover all views from a single view quite effectively from a visual inspection. Of course, simultaneous exposure to multiple views is not possible for human observers, therefore we are now exploring models with greater biological plausibility in which they are only exposed to one view at a time.

## Threshold versus Accumulator Frameworks of Manual Steering and Automation Takeover Initiation

**Courtney M Goodridge^1^, Jac Billington^1^, Gustav Markkula^1,2^, Callum D Mole^1^ and Richard M Wilkie^1^**

^1^School of Psychology, University of Leeds, Leeds, UK

^2^Institute for Transport studies, University of Leeds, Leeds, UK

pscmgo@leeds.ac.uk

Vehicle control is possible because the human nervous system is capable of producing complex sensorimotor actions. Drivers must monitor errors and initiate steering corrections of the correct magnitude and timing to maintain safe lane positions. The perceptual mechanisms determining how a driver processes visual information and initiates corrections remains unclear. Perceptual-motor action literature suggests two potential alternative mechanisms for responding to errors: (i) perceptual evidence (error) satisficing fixed constant thresholds (Threshold), or (ii) the integration of perceptual evidence over time (Accumulator). To distinguish between these mechanisms three experiments were conducted using steering correction paradigms. In the first two experiments, drivers (N = 20) steered towards intermittently appearing ‘road-lines’ that varied in position and orientation with respect to the driver’s starting position and trajectory. In the third experiment (N = 50), silent automation failures were induced at varying severities. Drivers had to disengage automation and initiate steering to avoid exceeding lane boundaries. Threshold and Accumulator accounts predicted different steering patterns responding to these errors: a Threshold account predicted fixed absolute error responses across conditions regardless of the rate of error development, whereas an Accumulator account predicted larger absolute error responses when the error signal developed faster. All three experiments show that drivers responded faster, responded to larger quantities of error, and steered with greater magnitude, as the rate of error signal development increased. These findings are in line with an Accumulator account, thus we propose that models of steering and silent failure takeovers should integrate perceptual evidence over time to capture human perceptual performance.

## A simple perceptual model of surface colours in naturalistic scenes: first assessments

**Hamed Karimipour and Christoph Witzel**

School of Psychology, University of Southampton, Southampton, UK

h.karimipour@southampton.ac.uk

While we can measure the colour of light (e.g., of a lamp or a computer screen), the colours of surfaces (such as the colours of objects and materials) depend on the interaction between the reflectance spectra of the surfaces and the spectra of the light shining on the surfaces. The perceptual effects of this spectral interaction can be approximated by a simple, linear transformation that is characteristic to the reflectance properties of each surface. In this study, we evaluated this approximation for naturalistic scenes under smooth, naturalistic and narrowband artificial lighting. Using a 4-Alternative-Forced-Choice discrimination task in an online study with 370 participants, we tested whether human observers can discriminate between the approximate and the spectral rendering of scenes. We rendered four scenes based on hyperspectral images, under 4 smooth and 4 narrow-band illuminants with colours along and orthogonal to the daylight locus. Results showed that participants responded clearly above chance level for images rendered with red and blue narrowband illuminants; however, they were at chance level with yellow and green narrowband, and with all smooth illuminants. The results with a few example scenes suggest that our simple approximation is well fit to characterise surface colours in the natural environment, but not when seen under narrowband, artificial illuminants. Additional measurements are planned to make sure these findings generalise hold with other scenes and are representative for the natural environment.

## Using vision tests for early detection of Alzheimer’s disease and related dementias

**Eugenie Roudaia^1^, Ali Hashemi^5^, Nicole D. Anderson^1,2,3^, Claude Alain^1,2^, Rosanne Aleong^1^, Nasreen Khatri^1^, Morris Freedman^1,4^ and Allison B. Sekuler^1,2,5^**

^1^Rotman Research Institute, Baycrest Health Sciences, Toronto, Canada

^2^Department of Psychology, University of Toronto, Toronto, Canada

^3^Department of Psychiatry, University of Toronto, Toronto, Canada

^4^Department of Medicine, University of Toronto, Toronto, Canada

^5^Department of Psychology, Neuroscience & Behaviour, McMaster University, Hamilton, Canada

eroudaia@research.baycrest.org

Alzheimer’s disease (AD) affects various aspects of visual processing, and these changes may be evident at the preclinical stage of mild cognitive impairment (MCI) that often precedes AD dementia. In this study, we evaluated 14 older adults diagnosed with MCI (mean age: 74.8 years) and 15 controls (mean age: 73.1 years) on a range of visual perceptual tasks while recording electroencephalography (EEG) using a consumer-grade device. Perceptual tests included discrimination of contours in clutter, face identification, optic flow, biological motion detection, and emotion recognition in biological motion. On average, the MCI group showed lower density thresholds in the contours in clutter test (p = 0.003, Hedges’ g = −1.26) and worse accuracy recognizing emotion in biological motion (p = 0.03, g = −1.08). There were no group differences in behavioural performance in the other measures. EEG data were analyzed by extracting the peak and amplitude of the first positive peak (P1) and first negative peak (N1) in each task. The latency of the P1 and N1 components was higher in the MCI group in the contours in clutter test (p = 0.009, g = 1.26), and for the P1 component in the face identification test (p = 0.045, g = 1.03). Scores on a measure of global cognition (Montreal Cognitive Assessment) was associated with thresholds in the contours task (rho = 0.46), emotion recognition accuracy (rho = 0.54), and the N1 latency in the contours in clutter (rho = −0.57), face identification (rho = −0.52), and optic flow tasks (rho = −0.48). Simple visual tests show promise as markers of preclinical AD.

## Visual-odour correspondences are partly explained by chemical features

**Ryan J Ward^1^, Sophie M Wuerger^2^ and Alan Marshall^1^**

^1^Department of Electrical & Electronic Engineering, University of Liverpool, Liverpool, UK

^2^Department of Psychology, University of Liverpool, Liverpool, UK

Ryan.ward@liverpool.ac.uk

Odour perception occurs when our olfactory receptors transduce volatile molecules. The bindings between our olfactory receptors are believed to recognise specific chemical features (Kermen & Chakirican et al., 2011 Nature Scientific Reports 206 1-6). However, the role the underlying chemical features plays in explaining olfactory crossmodal correspondences is still unknown. An artificial equivalent of the human olfactory stimulus (electronic nose) can uncover the underlying chemical features in the vapour phase. Here we show that the underlying chemical features contribute towards explaining olfactory crossmodal correspondences, namely, the visual dimensions (colour and the angularity of shapes) and other dimensions (smoothness of texture, pitch and perceived pleasantness). A Procrustes analysis revealed a reasonable degree of similarity (69%) between the chemical and perceptual spaces and that the perceptual features can be predicted/matched using the underlying chemical features. Overall these findings go against the common mediation hypotheses for olfactory crossmodal correspondences and suggest there is a predictable link between the underlying chemical features of the odours and the perception of the visual features.

